# Supplementary material for: Genetic diversity of Ralstonia solanacearum causing vascular bacterial wilt under different agro-climatic regions of West Bengal, India
Source: PLoS One. 2022 Sep 22;17(9):e0274780. doi: 10.1371/journal.pone.0274780 (PMC9498970; doi:10.1371/journal.pone.0274780)
Supplement: S1 Table — (DOCX) [file pone.0274780.s001.docx]

**S1 Table. Information of primers and PCR conditions used for amplification of genes for the conformation of *R. solanacearum* and MLSA study**

| **Gene** | **Amplicon size (bp)** | **Primers used** | **Sequences (5’-3’)** | **Temperature conditions in Thermocycler** | **References** |
| --- | --- | --- | --- | --- | --- |
| *Ralstonia solanacearum* species specific 16S rDNA regions | 288 | OLI1 | GGGGGTAGCTTGCTACCTGCC | Initial denaturation at 94°C for 2 min, 35 cycles consisting denaturation at 94°C for 20 sec, annealing at 68°C for 20 sec and extension at 72°C for 20 sec. Final extension of 72°C for 10 min was provided. | [1] |
|  |  | Y2 | CCCACTGCTGCCTCCCGTAGGAGT |  |  |
| 16S rDNA | 1500 | 27F | AGAGTTTGATCMTGGCTCAG | Initial denaturation at 95°C for 7 min, 35 cycles consisting denaturation at 94°C for 1 min, annealing at 52°C for 1 min and extension at 72°C for 8 min. Final extension of 72°C for 15 min was provided. | [2] |
|  |  | 1525R | AAGGAGGTGWTCCARCC |  |  |
| *egl* | 850 | EndoF | ATGCATGCCGCTGGTCGCCGC | Initial denaturation at 96°C for 9 min, 30 cycles of denaturation at 95°C for 1min, annealing at 70°C for 1 min and extension at 72°C for 2 min followed by a final extension step of 72°C for 10 min. | [3,4] |
|  |  | Endo R | GCGTTGCCCGGCACGAACACC |  |  |
| *hrp*B | 1417 | RShrpBf | TGCCATGCTGGGAAACATCT | Initial denaturation step of 95°C for 5 min, followed by 30 cycles of denaturation at 95°C for 30 sec, annealing at 64°C for 30 sec and extension at 68°C for 2 min. The final 20 cycles were the same as the first 10 with an additional 20 sec added to the elongation step for each new cycle and a final extension step at 68°C for 7 min. | [5] |
|  |  | RShrpBr | GGGGGCTTCGTTGAACTGC |  |  |
| *adk* | 536 | adk-Fa | TCTGTTGGGCGCACCCGGC | Initial denaturation at 95°C for 9 min, 35 cycles consisting denaturation at 95°C for 30 sec, annealing at 62°C for 1 min and extension at 72°C for 2 min. Final extension of 72°C for 10 min was provided. | [6] |
|  |  | adk-Rr | CCCAGCCGGAGTAGTAGTCC |  |  |
| *gyr*B | 432 | gyrB1F | GACAACGGCCGCGGSATTCC | Initial denaturation at 95°C for 9 min, 35 cycles consisting denaturation at 95°C for 30 sec, annealing at 54°C for 1 min and extension at 72°C for 2 min. Final extension of 72°C for 10 min was provided. | [7] |
|  |  | gyrB2R | CACGCCGTTGTTCAGGAASG |  |  |
| *gdh*A | 1056 | GdhAF | GATGGATGACGGCCGCATCG | Initial denaturation at 95°C for 9 min, 35 cycles consisting denaturation at 95°C for 30 sec, annealing at 63°C for 1 min and extension at 72°C for 2 min. Final extension of 72°C for 10 min was provided. | [[6]](#_ENREF_4) |
|  |  | GdhAR | TGAACGCCGCCGTCCGCAG |  |  |
| *leu*S | 793 | leuS27-F | GGCGCAGAAGGTCACGCCCA | Initial denaturation at 95°C for 9 min, 35 cycles consisting denaturation at 95°C for 30 sec, annealing at 59°C for 1 min and extension at 72°C for 2 min. Final extension of 72°C for 10 min was provided. | [8] |
|  |  | leuS819-R | GGCGCAGAAGGTCACGCCCA |  |  |
| *fli*C | 380 | Rsol *fliC*-F | GAACGCCAACGGTGCGAACT | Initial denaturation at 95°C for 9 min, 35 cycles consisting denaturation at 95°C for 30 sec, annealing at 63°C for 1 min and extension at 72°C for 2 min. Final extension of 72°C for 10 min was provided. | [9] |
|  |  | Rsol *fliC*-R | GGCGGCCTTCAGGGAGGTC |  |  |
| *pps*A | 1210 | ppsAN-F | GGGCGTGATGTTCACGAT | Initial denaturation at 95°C for 9 min, 35 cycles consisting denaturation at 95°C for 30 sec, annealing at 57°C for 1 min and extension at 72°C for 2 min. Final extension of 72°C for 10 min was provided. | [[6]](#_ENREF_4) |
|  |  | ppsANR | CCAGCATGGGGTTCTCTTC |  |  |

**References**

1. Seal SE, Jackson LA, Young JP, Daniels MJ. Differentiation of *Pseudomonas solanacearum, Pseudomonas syzygii, Pseudomonas pickettii* and the Blood Disease Bacterium by partial 16S rRNA sequencing: construction of oligonucleotide primers for sensitive detection by polymerase chain reaction. J Gen Microbiol. 1993;139(7): 1587-94. doi: 10.1099/00221287-139-7-1587. PMID: 8371118.
2. Poussier S, Trigalet-Demery D, Vandewalle P, Goffinet B, Luisetti J, Trigalet A. Genetic diversity of *Ralstonia solanacearum* as assessed by PCR-RFLP of the *hrp* gene region, AFLP and 16S rRNA sequence analysis, and identification of an African subdivision. Microbiology (Reading). 2000;146(7):1679-1692. doi: 10.1099/00221287-146-7-1679. PMID: 10878132.
3. Fegan M, Taghavi M, Sly LI, Hayward AC. Phylogeny, diversity and molecular diagnostics of Ralstonia solanacearum. In Bacterial Wilt Disease 1998 (pp. 19-33). Springer, Berlin, Heidelberg.
4. Fegan M, Prior P. How complex is the *Ralstonia solanacearum* species complex. *Bacterial Wilt Disease and the Ralstonia solanacearum species complex*. In: Allen C, Prior P, Hayward AC, editors. APS Press, St. Paul, M. N. 2005. pp.449–461.
5. Poussier S, Prior P, Luisetti J, Hayward C, Fegan M. Partial sequencing of the *hrp*B and endoglucanase genes confirms and expands the known diversity within the *Ralstonia solanacearum* species complex. Syst Appl Microbiol. 2000;23(4): 479-86. doi: 10.1016/S0723-2020(00)80021-1. PMID: 11249017.
6. Castillo JA, Greenberg JT. Evolutionary dynamics of *Ralstonia solanacearum*. Appl Environ Microbiol. 2007;73(4):1225-38. doi: 10.1128/AEM.01253-06. Epub 2006 Dec 22. PMID: 17189443; PMCID: PMC1828673.
7. Tayeb LA, Lefevre M, Passet V, Diancourt L, Brisse S, Grimont PAD. Comparative phylogenies of Burkholderia, Ralstonia, Comamonas, Brevundimonas and related organisms derived from *rpoB, gyrB* and *rrs* gene sequences. Res Microbiol. 2008;159(3):169–177. doi: 10.1016/j.resmic.2007.12.005. PMID: 18280706.
8. Wicker E, Lefeuvre P, de Cambiaire JC, Lemaire C, Poussier S, Prior P. Contrasting recombination patterns and demographic histories of the plant pathogen *Ralstonia solanacearum* inferred from MLSA. ISME Journal. 2012;6(5): 961-74. doi: 10.1038/ismej.2011.160. PMID: 22094345.
9. Schönfeld J, Heuer H, Van Elsas JD, Smalla K. Specific and sensitive detection of Ralstonia solanacearum in soil on the basis of PCR amplification of fliC fragments. Appl Environ Microbiol. 2003;69(12):7248-56. doi: 10.1128/AEM.69.12.7248-7256.2003. PMID: 14660373; PMCID: PMC309886.
