# Supplementary material for: Genetic diversity of Ralstonia solanacearum causing vascular bacterial wilt under different agro-climatic regions of West Bengal, India
Source: PLoS One. 2022 Sep 22;17(9):e0274780. doi: 10.1371/journal.pone.0274780 (PMC9498970; doi:10.1371/journal.pone.0274780)
Supplement: S2 Table — (DOCX) [file pone.0274780.s002.docx]

**S2 Table. Accession numbers of *R. solanacearum* partial gene sequences retrieved from NCBI**

| **Isolates** | **16S** | ***egl*** | ***hrp*B** | ***leu*S** | ***adk*** | ***gdh*A** | ***gyr*B** | ***fli*C** | ***ppsA*** |
| --- | --- | --- | --- | --- | --- | --- | --- | --- | --- |
| T1 | MN587258 | MN593217 | MT150907 | MN443133 | MK736269 | MN558701 | MN593226 | MN565697 | OM248664 |
| B2 | MK178506 | MN450428 | MT150908 | MN443118 | MN832424 | MN558702 | MN593227 | MN565698 | OM248665 |
| B5 | MK178507 | MN450429 | MT150909 | MN443134 | MK736270 | MN558703 | MN593228 | MN565699 | OM248666 |
| B8 | MK178508 | MN450430 | MT150910 | MN443135 | MK736271 | MN558704 | MN593229 | MN565700 | ON221416 |
| B9 | MK178509 | MN450431 | MT150911 | MN443136 | MK736272 | MN558705 | MN593230 | MN565701 | ON221417 |
| B10 | MK178510 | MN450432 | MT150912 | MN443119 | MK736273 | MN558706 | MN593231 | MN565702 | ON221418 |
| T11 | MK178511 | MN450433 | MT150913 | MN443137 | MN832425 | MN558707 | MN593232 | MN565703 | ON221419 |
| B12 | MK178512 | MN450434 | MT150914 | MN443120 | MN420515 | MN558708 | MN593233 | MN566919 | ON221420 |
| B13 | MK178513 | MN450435 | MT150915 | MN443138 | MN832426 | MN558709 | MN593234 | MN565704 | ON221421 |
| T14 | MK178514 | MN450436 | MT150916 | MN443139 | MN420516 | MN558710 | MN593235 | MN565705 | ON221422 |
| B15 | MK178515 | MN450437 | MT150917 | MN593262 | MN420517 | MN558711 | MN593236 | MN565706 | ON221423 |
| B16 | MK178516 | MN450438 | MT150918 | MN443140 | MN443125 | MN558712 | MN593237 | MN565707 | ON221424 |
| B17 | MN587259 | MN450439 | MT150919 | MN443121 | MN443126 | MN558713 | MN593238 | MN565708 | ON221425 |
| T18 | MN587260 | MN450440 | MT150920 | MN443141 | MN832427 | MN558714 | MN593239 | MN565709 | ON221426 |
| B19 | MN587261 | MN593218 | MT150921 | MN593263 | MT022088 | MN558715 | MN593240 | MN565710 | ON221427 |
| C20 | MN587262 | MN450441 | MT150922 | MN443142 | MN832428 | MN558716 | MN593241 | MN565711 | ON221428 |
| T21 | MT138812 | MN450442 | MT150923 | MN443143 | MN443127 | MN558717 | MN593242 | MN565712 | ON221429 |
| Bo22 | MN587263 | MN450443 | MT150924 | MN443122 | MN832429 | MN558718 | MN593243 | MN565713 | ON221430 |
| Ca23 | MN587264 | MN450444 | MT150925 | MN443144 | MN832430 | MN558719 | MN593244 | MN565714 | ON221431 |
| T24 | MN587265 | MN450445 | MT150926 | MN443145 | MN832431 | MN558720 | MN593245 | MN566920 | ON221432 |
| B26 | MK178518 | MN450446 | MT150927 | MN443146 | MN832432 | MN558721 | MN593246 | MN565715 | ON221433 |
| B27 | MK178519 | MN450447 | MT150928 | MN443147 | MN420518 | MN558722 | MN593247 | MN565716 | ON221434 |
| B28 | MK178520 | MN593219 | MT150929 | MN443148 | MN443128 | MN558723 | MN593248 | MN565717 | ON221435 |
| B30 | MT138813 | MN593220 | MT150930 | MN443149 | MN832433 | MN558724 | MN593249 | MN565718 | ON221436 |
| B32 | MK178523 | MN450448 | MT150931 | MN443150 | MN832434 | MN558725 | MN593250 | MN565719 | ON221437 |
| B34 | MK178524 | MN593221 | MT150932 | MN443151 | MN832435 | MN558726 | MN593251 | MN565720 | ON221438 |
| M36 | MK178525 | MN593222 | MT150933 | MN443152 | MN832436 | MN558727 | MN593252 | MN565721 | ON221439 |
| B39 | MN587266 | MN450449 | MT150934 | MN443153 | MN443129 | MN558728 | MN593253 | MN565722 | ON221440 |
| B41 | MN587267 | MN593223 | MT150935 | MN443154 | MN832437 | MN558729 | MN593254 | MN565723 | ON221441 |
| B42 | MN587268 | MN450450 | MT150936 | MN593264 | MN832438 | MN558730 | MN593255 | MN565724 | ON221442 |
| B43 | MN587269 | MN450451 | MT150937 | MN443123 | MN832439 | MN558731 | MN593256 | MN565725 | ON221443 |
| B44 | MN587270 | MN593224 | MT150938 | MN443124 | MN443130 | MN558732 | MN593257 | MN565726 | ON221444 |
| B45 | MN587271 | MN593225 | MT150939 | MN443155 | MN420519 | MN558733 | MN593258 | MN565727 | ON221445 |
| B50 | MN587272 | MN450452 | MT150940 | MN443156 | MN443131 | MN558734 | MN593259 | MN565728 | ON221446 |
| C51 | MN587273 | MN450451 | MT150941 | MN443157 | MN443132 | MN558735 | MN593260 | MN565729 | ON221447 |
| C52 | MN587274 | MN450452 | MT150942 | MN443158 | MN832440 | MN558736 | MN593261 | MN565730 | ON221448 |
