# Supplementary material for: Genetic diversity of Ralstonia solanacearum causing vascular bacterial wilt under different agro-climatic regions of West Bengal, India
Source: PLoS One. 2022 Sep 22;17(9):e0274780. doi: 10.1371/journal.pone.0274780 (PMC9498970; doi:10.1371/journal.pone.0274780)
Supplement: S2 Fig — (DOCX) [file pone.0274780.s005.docx]

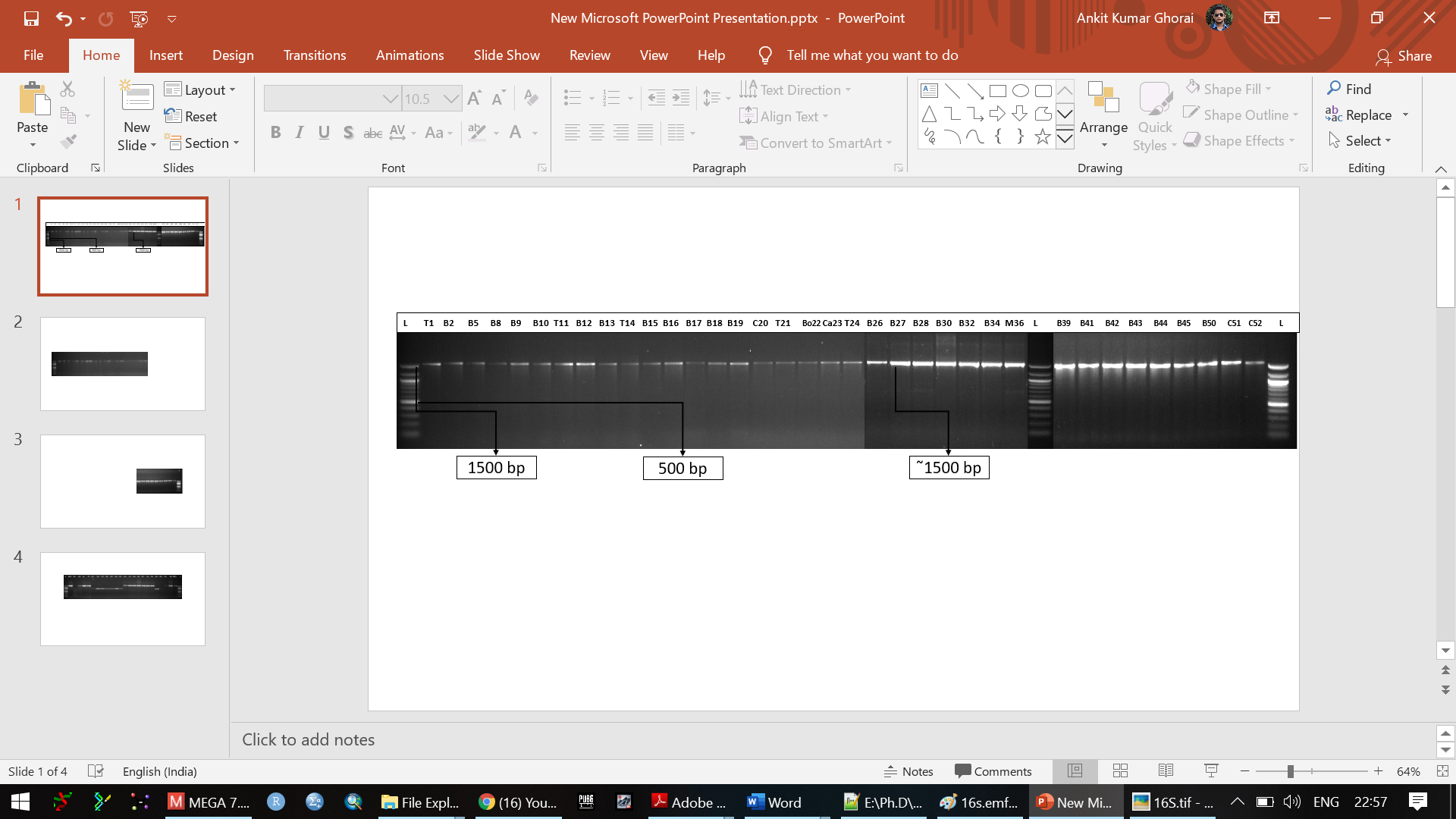


**S2 Fig. Amplification of 36 isolates of *R. solanacearum* producing amplicon of approximately 1500 bp of the 16S rDNA regions with 27F-1525R primer pairs in 0.8 % agarose gel. Lane L: 100 bp ladder.**
