# Supplementary material for: Genetic diversity of Ralstonia solanacearum causing vascular bacterial wilt under different agro-climatic regions of West Bengal, India
Source: PLoS One. 2022 Sep 22;17(9):e0274780. doi: 10.1371/journal.pone.0274780 (PMC9498970; doi:10.1371/journal.pone.0274780)
Supplement: S3 Fig — (DOCX) [file pone.0274780.s006.docx]

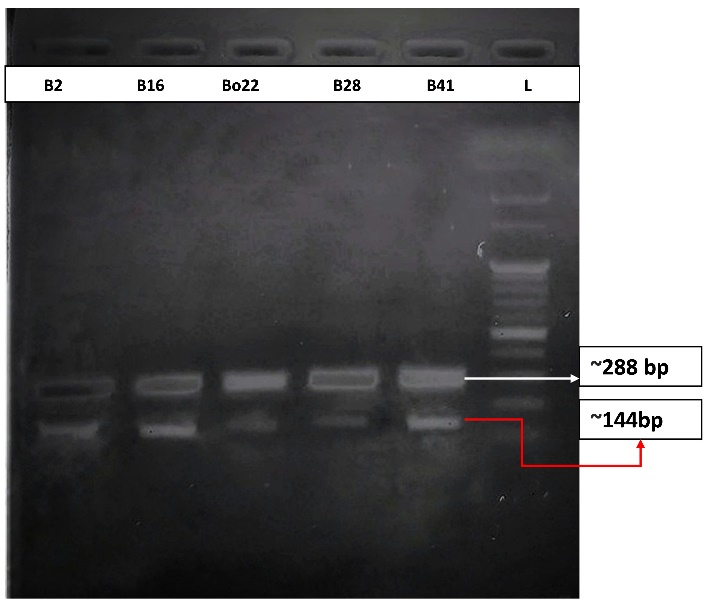


**S3 Fig. Gel electrophoresis of phylotype specific multiplex PCR for six isolates of *R. solanacearum* collected from various ACZ of West Bengal. The isolates produced amplicon of 144 bp specific to Phylotype I and 288 bp specific to *R. solanacearum*.**
