# Supplementary material for: Genetic diversity of Ralstonia solanacearum causing vascular bacterial wilt under different agro-climatic regions of West Bengal, India
Source: PLoS One. 2022 Sep 22;17(9):e0274780. doi: 10.1371/journal.pone.0274780 (PMC9498970; doi:10.1371/journal.pone.0274780)
Supplement: S5 Fig — (DOCX) [file pone.0274780.s008.docx]

**S5 Fig. Partial amplification of *hrp*B gene with 36 *R. solanacearum* isolates with *RShrpBf - RShrpBr* primer pairs producing approximately 1417 bp fragment. Lane L: 100 bp ladder.**
