# Supplementary material for: Genetic diversity of Ralstonia solanacearum causing vascular bacterial wilt under different agro-climatic regions of West Bengal, India
Source: PLoS One. 2022 Sep 22;17(9):e0274780. doi: 10.1371/journal.pone.0274780 (PMC9498970; doi:10.1371/journal.pone.0274780)
Supplement: Attachment — Submitted filename: Response to Reviewers.docx [file pone.0274780.s009.docx]

| **Line NO** | **Comments** | **Revision Done** |
| --- | --- | --- |
| 22 | Diseases can be corrected to disease | Corrected to “Disease” |
| 23 | (smith 1896) yabuuchi et al. 1995 citation may not be required in the abstract | Removed from abstract |
| 89 | “reacting positive” change wording | Replaced with “Isolation was done from plants with foliar epinasty or wilt symptoms after confirmation with ooze test” |
| 152 | “32 ±1°C or 27 ±1°C day/night” are the authors want to mean day temperature 32 ±1°C and 27 ±1°C as night temperature? Clarify and if necessary modify. | Replaced with “28-30 °C with 12 hours light and dark cycle and” |
| 164 | “Purification of extracted DNA was made by centrifugation at 15493g for 5 min” I think better not to refer the process as purification since it is just a simple centrifugation step. | Replaced and modified with “… followed by centrifugation at 15493g for 5 min” |
| 194 | “16S-23SITS”, after 23s give a space | Space provided |
| 317 | Pathogenecity test- Among 36 isolates, results of all the isolates are not mentioned properly. Please rectify. | The pathogenicity grouping on basis of pathogenicity test have been clarified for all 36 isolates under “pathogenicity test” heading and supporting data of pathogenicity has been provided in Table 3 under “PG” head. |
| 462 | “No values were obtained for non-synonymous selection” is it appropriate? | Changes were incorporated in the appropriate place.(Table 4 and line number 466 onwards). The value for K_a_ was observed for *adk*, *egl* and *pps*A whereas d_N_ was obtained for *adk*, *egl*, *gdh*A, *pps*A and the concatenated sequence. Thus, non-synonymous substitutions (represented by K_a_ and d_N_) were not observed for all the genes under consideration*.* However, except *leu*S, synonymous substitutions (represented by K_s_ and d_S_) were observed for all the other genes and the concatenated sequence. So absence of non-synonymous substitution indicates existence of purifying selection pressure. |
| 737 | Now in Line no: 750-752This Red and Lateritic Zone of West  Bengal shares the major border with solanaceous vegetable growing neighbouring state  Jharkhand, India.” Rewrite the sentence. | Line no: 750-752: Replaced with “This Red and Lateritic Zone of West Bengal (ACZ 6) shares border with the Jharkhand state, where solanaceous vegetable crops are widely cultivated.” |
|  | We note that [Figure 14] in your submission contain [map/satellite] images which may be copyrighted. All PLOS content is published under the Creative Commons Attribution License (CC BY 4.0), which means that the manuscript, images, and Supporting Information files will be freely available online, and any third party is permitted to access, download, copy, distribute, and use these materials in any way, even commercially, with proper attribution. For these reasons, we cannot publish previously copyrighted maps or satellite images created using proprietary data, such as Google software (Google Maps, Street View, and Earth). For more information, see our copyright guidelines: <http://journals.plos.org/plosone/s/licenses-and-copyright>. | The figure has already been published under CC BY 4.0 license by Pranab 2018 on July 4, 2001. The link is given below. <https://commons.wikimedia.org/wiki/File:AGRO-CLIMATE.WBpp.jpg>  Appropriate credit has been provided to the author of the original picture under caption of the figure 14 as per guidelines. |
